# Genetic diversity of laboratory strains and implications for research: The case of *Aedes aegypti*

**DOI:** 10.1371/journal.pntd.0007930

**Published:** 2019-12-09

**Authors:** Andrea Gloria-Soria, John Soghigian, David Kellner, Jeffrey R. Powell

**Affiliations:** Department of Ecology and Evolutionary Biology, Yale University, New Haven, United States of America; University of California, Davis, UNITED STATES

## Abstract

The yellow fever mosquito (*Aedes aegypti*), is the primary vector of dengue, Zika, and chikungunya fever, among other arboviral diseases. It is also a popular laboratory model in vector biology due to its ease of rearing and manipulation in the lab. Established laboratory strains have been used worldwide in thousands of studies for decades. Laboratory evolution of reference strains and contamination among strains are potential severe problems that could dramatically change experimental outcomes and thus is a concern in vector biology. We analyzed laboratory and field colonies of *Ae*. *aegypti* and an *Ae*. *aegypti*-derived cell line (Aag2) using 12 microsatellites and ~20,000 SNPs to determine the extent of divergence among laboratory strains and relationships to their wild relatives. We found that 1) laboratory populations are less genetically variable than their field counterparts; 2) colonies bearing the same name obtained from different laboratories may be highly divergent; 3) present genetic composition of the LVP strain used as the genome reference is incompatible with its presumed origin; 4) we document changes in two wild caught colonies over ~16 generations of colonization; and 5) the Aag2 *Ae*. *aegypti* cell line has experienced minimal genetic changes within and across laboratories. These results illustrate the degree of variability within and among strains of *Ae*. *aegypti*, with implications for cross-study comparisons, and highlight the need of a common mosquito repository and the implementation of strain validation tools.

## Author summary

Laboratory colonies provide the opportunity to study live organisms in a controlled environment and serve as phenotypic surrogates of their natural populations. Over time, these strains are prone to change as they face novel environments. We analyzed laboratory and field colonies of the yellow fever mosquito (*Aedes aegypti*), primary vector of dengue, Zika, and chikungunya fever and a model system in vector biology, and an *Ae*. *aegypti*-derived cell line (Aag2) to determine genetic similarity between laboratory strains and their wild relatives. We found lower levels of genetic diversity in laboratory populations compared to wild populations, with colonies of the same name diverging over time or likely contaminated. We also found that the genetic composition of the Liverpool strain, used as the reference genome for this species, is inconsistent with historical records that suggest an African origin and instead points to an outside Africa source. Finally, we did not find major genetic changes in Aag2 cell lines across laboratories. Laboratory evolution of reference strains and strain contamination are severe problems that can change experimental outcomes and complicate cross-study comparison. Our results illustrate the need of a common *Aedes aegypti* repository and the development of strain validation tools.

## Introduction

In nature, organisms live in complex environments. The inability of the researcher to control the surrounding physical and biological parameters in natural settings presents a challenge for many types of study. This problem can be overcome by bringing the research subject into the laboratory, where many, sometimes most, variables can be controlled and manipulated. Laboratory-reared individuals provide additional advantages, such as the availability of an unlimited supply of individuals of different sex and age, and a relatively homogeneous genetic background that reduces variability in research outcomes. Often, laboratory colonies become the reference for studies across research groups and get distributed among labs, or commercialized for use as “standards”. These reference strains evolve like any other living organism in response to an artificial environment [1–3], which usually involves constant optimal environmental conditions, sufficient food, easy access to mates, and specific propagation methods. Furthermore, every time a reference strain moves from one laboratory to another, it faces a novel environment (bottlenecks, feeding regimes, temperature fluctuations, etc.), and thus will tend to drift away from the original source [4]. For some model systems, such as microbes and certain nematodes [5], such change can be avoided or minimized by keeping frozen stocks of the original source. However, this is not an option for many organisms, and laboratory colonies need to be constantly bred in the laboratory to keep them viable [6,7]. Laboratory evolution of reference strains and contamination among strains are severe problems, as they can dramatically change the experimental outcomes of a study [8–11]. Starting in 2015, in an attempt to increase the reproducibility of research findings, the National Institutes of Health (NIH, USA) has required that all grant applications include a section entitled “Authentication of Key Biological and/or Chemical Resources” (NIH 2015 NOT-OD-15-011 and 012).

*Aedes aegypti* is an extensively studied mosquito in the laboratory [12–14] and the most popular laboratory model system for arthropod disease vectors due to its relative ease of rearing and manipulating in the lab. Established laboratory strains have been used in thousands of studies. The Rockefeller strain, ROCK, is probably the most widely used strain and its origin dates back to more than 100 years ago [15]. The Liverpool strain (LVP) was used to generate the entire genome sequence that is now used as the reference genome for the species [16, 17]. This strain was collected in “West Africa” and has been maintained at the Liverpool School of Tropical Medicine since 1936 [18].

We are conducting an ongoing global survey of genetic variation among and within *Aedes aegypti* populations ([19] and references therein). In the context of this work, we felt it important to determine how the genetic diversity of common *Ae*. *aegypti* laboratory strains compares to that now found in natural populations of *Ae*. *aegypti*. All of our recent work has been on mosquitoes derived directly from field collections (most often eggs or larvae) or, in a minority of cases, after one or two generations of laboratory rearing. Our samples were collected from 2004 onward and genotyped for both microsatellites and ~25,000 genome-wide SNPs [20], to survey genetic diversity in more than 200 samples from 38 countries on six continents (data publicly available through *VectorBase*.*org* [21]).

Here we ask: How do common laboratory strains compare genetically to natural populations? Is the level of genetic diversity different in laboratory strains compared to field populations? Can we assign lab strains to either of the recognized subspecies? Do accessions from different laboratories of the presumed same strain differ genetically? We also monitored genetic changes during ~16–17 generations of colonization of two field collections from Vietnam. Here, “strain” refers to a living *Ae*. *aegypti* colony that bears the same name in different laboratories, is thought to have a single origin, and assumed to have uniform phenotypic and genotypic characteristics. Since tissue cultures of *Ae*. *aegypti* cells are used in research, we have also included them in the analysis and call “accession” a sample from the same named cell line (Aag2) that has been independently obtained from different laboratories.

## Materials and methods

### *Aedes aegypti* datasets

Two different datasets of genetic markers were used in this study: a) twelve previously published microsatellite loci [22,23] and (b) a panel of ~25,000 SNPs [20]. Genotypes from all wild mosquito collections used in this study have been reported elsewhere [19, 23–26] and are available through VectorBase (*www*.*vectorbase*.*org* [21]).

### Mosquito collections, DNA extraction and genotyping

Microsatellite data from 1,423 individual *Aedes aegypti* mosquitoes and SNP data from 320 mosquitoes were used in this study. Description of the *Ae*. *aegypti* laboratory strains and cell lines used are described in S1 File. Samples from *Ae*. *aegypti* colonies were received as adult mosquitoes in 70–100% ethanol, or eggs on oviposition papers. Eggs were hatched at the Yale School of Epidemiology and Public Health insectary, reared to adults, and preserved in 100% ethanol at -20°C until DNA extraction. Five different accessions from the *Ae*. *aegypti* cell line Aag2 were received as frozen cell pellets or in fresh culture from which cells were collected for subsequent processing. DNA extraction and microsatellite genotyping was performed as described in [19]. Due to the excess of null alleles found at the B2 microsatellite locus in the Hanoi strain, and because the locus became monomorphic at generation four, this locus was excluded to study the effect of colonization in both Vietnam populations. SNP genotyping was conducted using the Axiom_aegypti1 SNP-chip (Life Technologies Corporation CAT#550481; [20]), as described in [26]. Only the first and last generations from the Vietnam strains were genotyped for SNPs.

Raw microsatellite calls are included in the S2 and S3 Files. Both microsatellite and SNP genotypes are available through Vector Base [21] popBio project VBP0000452.

### Genetic diversity and population genetic analyses

Average observed (H_o_) and expected (H_e_) heterozygosities were estimated in the GenAlEx v. 6.5 package [27]. The average number of alleles across loci or allelic richness (AR) and the number of unique alleles in a population or private allelic richness (PAR), were calculated in HPRARE v.1.1[28], which uses rarefaction to correct for unequal sample sizes. The non-parametric Kruskal Wallis test was used to detect significant differences in H_o_ and allelic richness between different groups of populations. Effective population size (Ne) was estimated from a single population sample (as opposed to sampling a population multiple times) using the bias-corrected version of the linkage disequilibrium (LD) method from Waples and Do [29], as implemented in NeEstimator v.2.0 [30]. Ne was estimated from temporal samples from Vietnam also in NeEstimator v. 2.0 [30] using the Waples (1989 [31]) method and three options for computing the standardized variance in allele frequency, F [F_e_ (Nei & Tajima 1981 [32]); F_k_ (Pollak 1983 [33]); and F_s_ (Jorde & Ryman 2007)[34]]. First degree relatives within a population were identified in the SNP dataset with the VCFtools 0.1.14 [35]—relatedness2 command, and subsequently removed with PLINK v.1.9. ([36] www.cog-genomics.org/plink/1.9/) to evaluate the effect of relatives on Ne estimates and genetic clustering; Ne from only those populations with more than 6 individuals remaining were subsequently used for the comparison. The pairwise genetic distances (Fst) between population pairs were calculated using the StAMPP v.1.5.1 package [37] implemented in R v. 3.4.0 [38]. Analysis of molecular variance (AMOVA) on allele frequencies within and between populations of the main laboratory colonies was performed with GenoDive v. 2.0b.27 [39].

Population structure was evaluated from the microsatellite dataset via the Bayesian clustering method implemented by the software STRUCTURE v. 2.3 [40], which identifies genetic clusters and assigns individuals to these clusters with no *a priori* information of sample location. The most likely number of clusters (K) was determined by conducting 20 independent runs from each K = 1 to 28. Each run assumed an admixture model and correlated allele frequencies using a burn-in value of 100,000 iterations followed by 500,000 repetitions. The optimal number of K clusters was determined following the guidelines of Prichard et al. [40] and the Delta K method [41], as implemented by STRUCTURE HARVESTER v.0.6.94 [42]. Results were plotted with the program DISTRUCT v.1.1 [43]. Principal component analysis and structure-like analyses based on sparse non-negative matrix factorization on the SNP dataset were conducted with LEA v.1.8.1 [44] available for R v. 3.4.0 [38], using 24–25 random individuals from each collection.

The program BOTTLENECK v. 1.2.02 was employed to determine whether a population exhibits a significant number of loci with an excess of heterozygotes, which may indicate a recent bottleneck event [45,46]. The program was run with the Infinite Allele Model (IAM); the Two-phase mutation model (T.P.M.) with a variance of the geometric distribution = 0.36, appropriate for most microsatellites as suggested by the authors; and the Stepwise Mutation Model (SMM). Significance of the bottleneck was assessed by the "sign test" and "Wilcoxon sign-rank test" implemented by the software.

Genetic assignment tests on the Aag2 cell lines against a dataset that included all laboratory colonies and wild populations included in this paper, were performed in GeneClass v.2.0 [47] using only the SNP data, since previous studies have shown higher accuracy of assignment using SNPs rather than microsatellites [24,48]. Ten independent runs were conducted with sets of ~3,276 SNPs drawn at random using the command—thin 0.2 from PLINK v.1.9. ([36] www.cog-genomics.org/plink/1.9/), and the Bayesian criteria for likelihood estimation to determine the population-assignment ranking [49]. Similarity between the Aag2 cell lines was evaluated with the Genotype Concordance tool from Picard v.2.18.16 [50] using the SNP dataset.

### Evidence of admixture and phylogenetic reconstruction

We evaluated whether laboratory colonies showed evidence of admixture using a three-population [F3] test [51], as implemented by TreeMix v.1.13 [52]. The F3 test is a t-test of the form A:B,C, where a significant negative value of the test statistic implies that population A is admixed from parent populations B and C. The resulting p-values were subsequently corrected with both Bonferroni and Holms corrections for multiple comparisons. TreeMix v. 1.13 [52] was then used to estimate the maximum likelihood topology from population allele frequencies from aforementioned SNP data, using default settings and one final global rearrangement after all populations had been added (flag -global). To assess support for the maximum likelihood topology, TreeMix v. 1.13 [52] was used to generate 100 bootstrap replicates (-bootstrap flag), and the resulting bootstrapped trees were summarized on the full dataset topology using SumTrees from DendroPy v.4.4.0 [53], and visualized in FigTree v.1.4.4 (available from http://tree.bio.ed.ac.uk/software/figtree/).

## Results

### Laboratory colonization reduces genetic diversity in *Ae*. *aegypti*

**Fig 1:**
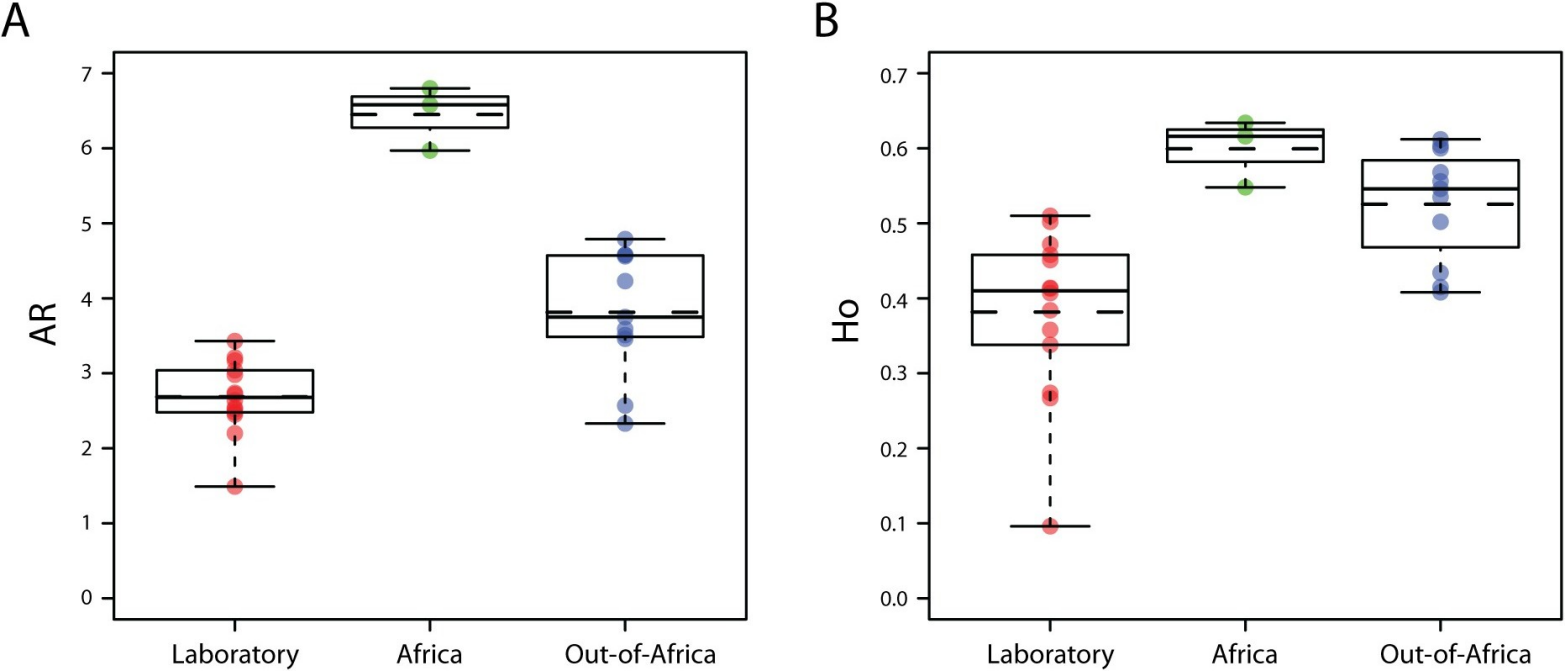
Genetic diversity of laboratory strains relative to representative wild populations of *Aedes aegypti* based on 12 microsatellites. A) Allelic Richness [AR] and B) Heterozygosity [Ho]. Each dot represents a population sample. Green: Africa, blue: outside Africa, red: laboratory strains. Boxplot show median (continuous line) and mean (dotted line) along with the 1st and 3rd quartile, whiskers identify the minimum and maximum values.

Across 12 multi-allelic microsatellite loci, the average allelic richness (AR) estimated from *Ae*. *aegypti* laboratory colonies is lower than that of wild collections (2.69 ± 0.4878 and 4.38 ±1.3482 respectively; H_1_ = 12.848, p = 0.0003; Africa: 6.45 and Out-Africa: 3.81); Table 1 and Fig 1A. Likewise, observed heterozygosity (Ho) is lower in laboratory colonies than in wild collections (0.3817 ± 0.1114 and 0.5413 ± 0.0756; H_1_ = 13.347, p = 0.0002; Africa: 0.5993 and Out-Africa: 0.5254); Table 1 and Fig 1B. These same parameters were measured across generations of laboratory rearing of two Vietnam populations from the initial colonization event. We observed a decline of overall AR with every generation sampled (F_[1]_ = 7.958, p = 0.0257), influenced by the geographic origin of the population (F_[1]_ = 9.249, p = 0.0188). However, no relationship was observed between the number of generations in the lab and the reduction in heterozygosity (F_[1]_ = 0.376, p = 0.559) (S1 Table).

**Table 1:** Diversity of laboratory and wild *Ae*. *aegypti* strains based on 12 microsatellites.

Population	N	Origin	Ho	uHe	AR(48)	PAR(48)
Hamburg_Strain	54	Lab	0.451	0.471	2.64	0
ROCK_Notre Dame	54	Lab	0.358	0.366	2.45	0
ROCK_FC	53	Lab	0.458	0.497	3.43	0
ROCK_Hopkins	54	Lab	0.338	0.372	2.52	0
Oxitec_513A	54	Lab	0.414	0.464	2.74	0
LVP_AagL1	53	Lab	0.096	0.087	1.49	0
LVP_WRAIR	54	Lab	0.413	0.46	2.98	0.01
Bangkok Strain	54	Lab	0.267	0.277	2.48	0.14
D2S3_WRAIR	54	Lab	0.502	0.486	2.72	0.04
Chetumal Strain	54	Lab	0.407	0.408	3.21	0.03
ORL_CAES	54	Lab	0.384	0.389	2.54	0
Surabaya Strain	54	Lab	0.274	0.278	2.2	0.04
Hanoi Strain	48	Lab	0.51	0.537	3.04	0
Ho Chi Minh Strain	50	Lab	0.472	0.456	3.17	0.03
Key West, FL, USA	52	Wild	0.612	0.615	4.58	0.02
Hanoi, VT	54	Wild	0.434	0.473	3.46	0
Ho Chi Minh, VT	54	Wild	0.568	0.583	4.79	0.12
New Orleans, LA,USA	24	Wild	0.604	0.633	4.58	0
Tapachula, MX	54	Wild	0.502	0.503	3.51	0.01
Chetumal, MX	54	Wild	0.535	0.498	3.59	0
Siquirres, CR	50	Wild	0.546	0.612	4.23	0.01
Yaounde, CM	54	Wild	0.548	0.635	5.97	0.34
Lope Forest, GA	54	Wild	0.634	0.71	6.8	0.61
Cairns, AU	24	Wild	0.556	0.596	3.75	0.33
Ouagadougou, BF	54	Wild	0.616	0.699	6.58	0.42
Bangkok, TH	50	Wild	0.415	0.4	2.57	0
Houston, TX, USA	47	Wild	0.408	0.374	2.33	0
Patillas, PR	54	Wild	0.6	0.6	4.56	0.05

*N* = sample size; Ho: observed heterozygosity, uHe: unbiased expected heterozygosity; AR and PAR: allelic richness and private allelic richness estimated by rarefaction (N = 48 genes).

**Fig 2:**
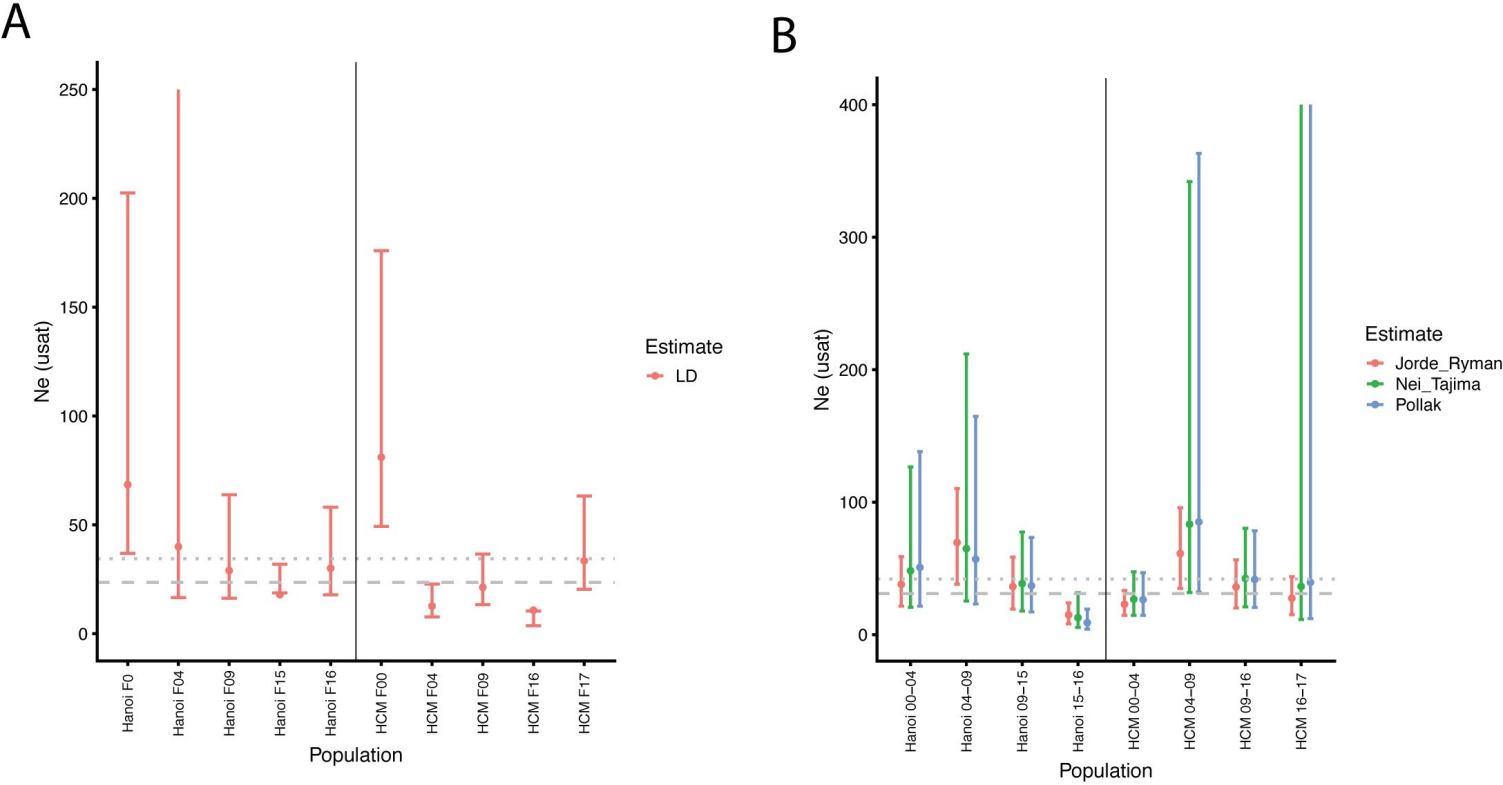
Effective population size (Ne) based on 12 microsatellites, estimated from A) a single-population sample using the bias-corrected version of the linkage disequilibrium method (LD) from [29], as implemented in NeEstimator v.2.0 [30] and B) population pairs using the two-sample Waples (1989 [31]) method and three options for computing the standardized variance in allele frequency, F [F_e_ (Nei & Tajima 1981[32]); Fk (Pollak 1983[33]); and Fs (Jorde & Ryman 2007)[34]]. Both methods implemented in NeEstimator v.2.0 [30]. Dotted lines are the arithmetic mean; dashed lines are the harmonic means.

The effective population size (Ne) estimated for the laboratory strains was not different from that of the wild collections, regardless of the genetic markers used in the estimations (S2 and S3 Tables) or whether first degree relatives were removed from the SNP dataset (S4 Table). Based on single population samples, microsatellite mean Ne_LAB_ = 67.44 ± 51.06 and Ne_WT_ = 38.94 ± 29.03; H_1_ = 0.21118, p = 0.6458) with the estimates displaying a large margin of error (S1A Fig and S2 Table). Equivalent SNP-based estimates yield a mean Ne_LAB_ = 15.12 ± 12.44 and Ne_WT_ = 32.01 ± 34.78; H_1_ = 1.2256, p = 0.2683 (S1B Fig and S3 Table). After first-degree relatives were removed, SNP-based estimates yield a mean Ne_LAB_ = 22.68 ± 11.37 and Ne_WT_ = 47.94 ± 42.21; H_1_ = 1.7455, p = 0.1864 (S4 Table). When Ne was calculated across generations (Hanoi and HCM), associated errors were reduced using the two-population method (temporal method applied to generation pairs), relative to the single-population method (Fig 2 and S5 and S6 Tables). Analysis of variance on these data suggests that Ne is affected by the generations spent in the laboratory and not by the geographic population of origin (F_[1]_ = 5.774, p = 0.0473; S7 Table), with Ne being larger at the beginning of the colonization process and decaying over time (H_1_ = 4.3636, p = 0.0367); see Fig 2 and S6 Table. Bottlenecks were detected during the colonization of HCM at generations F9, F16, and F17 with the IAM. In contrast, evidence of bottlenecks during the Hanoi colonization were detected at generation F4, F9, F15, and F16, using IAM, with bottlenecks at F15 and F16 further supported by the TPM and SMM (S8 Table).

Taken together, these data point to a significant and rapid loss of wild alleles upon laboratory colonization, likely a consequence of the stochasticity during the process, aided by fluctuations in the Ne at each generation.

### Strains with the same name may differ and do not represent the wild population from which they are thought to have originated

**Fig 3:**
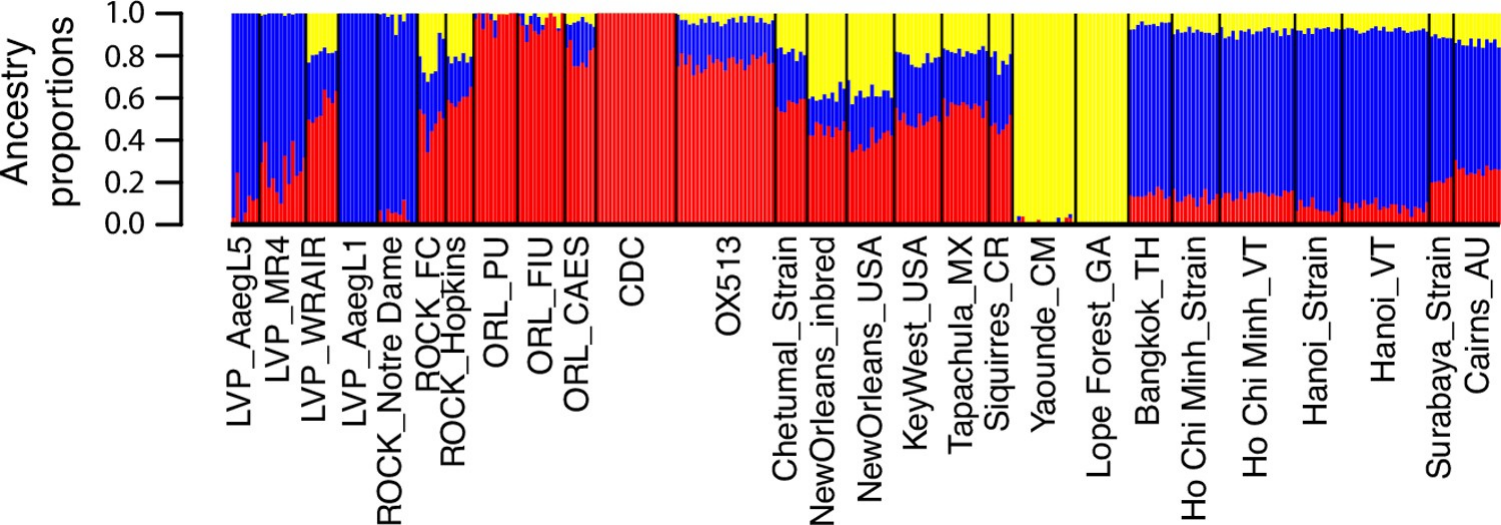
Genetic structure present among *Ae*. *aegypti* laboratory and wild strains. LEA v.1.8.1 [44] admixture bar plots based on 16,204 SNPs. Each vertical bar represents an individual. The height of each bar represents the probability of assignment to each of K = 3 genetic clusters (different colors). Rockefeller (ROCK); Orlando (ORL), Liverpool (LVP).

**Fig 4:**
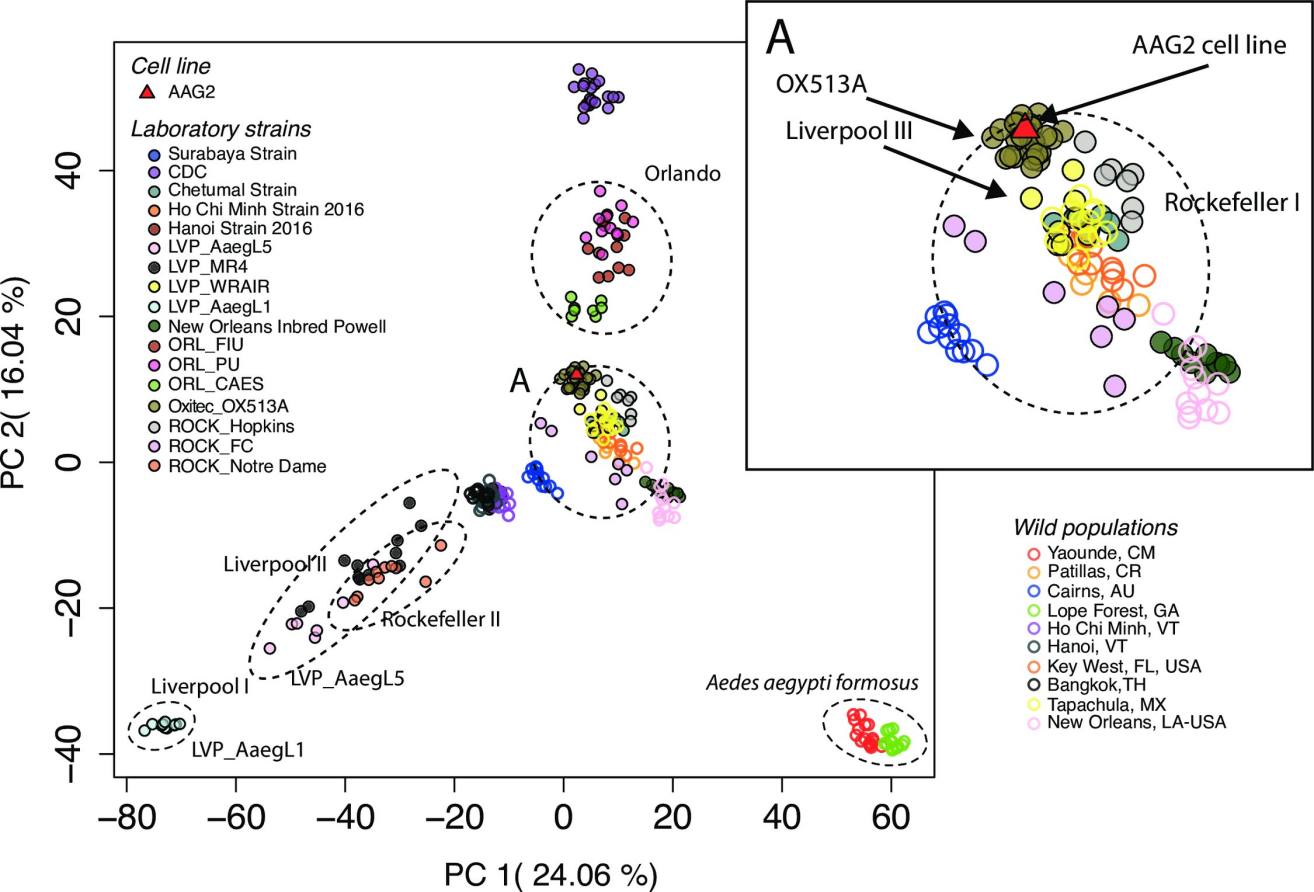
Principal component analysis (PCA) of 16,204 SNPs including *Aedes aegypti* wild populations (outlined circles), laboratory colonies (filled circles), and the Aag2 cell lines (triangles). Each population/colony is represented by a different color. Rockefeller (ROCK); Orlando (ORL), Liverpool (LVP).

**Fig 5:**
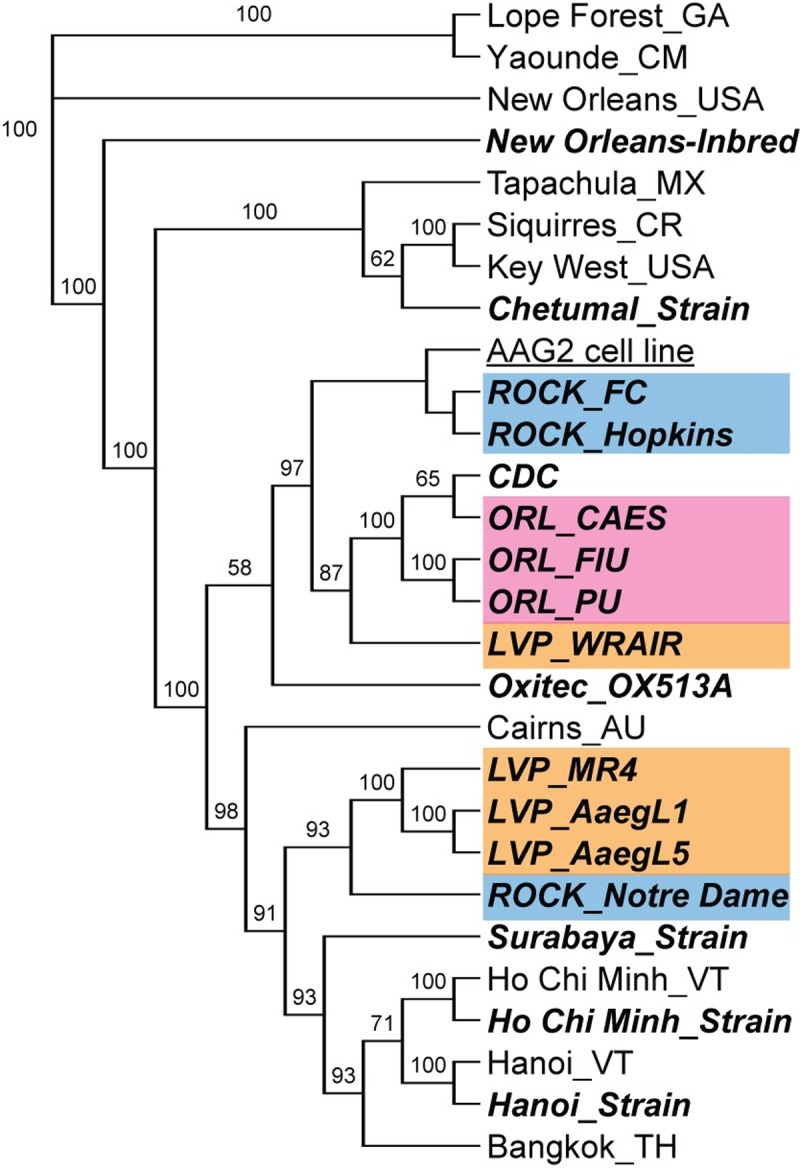
Cladogram of laboratory and wild strains of *Aedes aegypti* based on 16, 204 SNPs showing the topology of the maximum likelihood tree. Support values are reported above the branches. Branch length is not informative. Laboratory strains are in bold and italics. The *Ae*. *aegypti* cell line is underlined. Rockefeller (ROCK- blue); Liverpool (LVP- orange); Orlando (ORL- pink).

Population differentiation among colonies of the Rockefeller strain (ROCK), as well as of the Liverpool strain (LVP) was evident in the genetic clustering analysis on both the SNP and the microsatellite dataset, with strains from different labs belonging to different major genetic clusters (Figs 3 and S2). Results from the same analysis on the SNP dataset after removal of first-degree relatives is shown in S3 Fig (see Methods). Such differentiation was also observed in the SNP-based PCA (Fig 4) and on the Maximum Likelihood tree built from the same SNP dataset (Fig 5), where these strains are spread across different genetic groups or major clades, respectively. This pattern was not observed in the Orlando strain (ORL), which is more cohesive. Genetic differentiation (Fst) estimated within the ROCK and LVP strains was similar to the differentiation estimated among the ROCK, LVP, and ORL strain groups, with genetic differentiation among different sources of the ORL strain being considerably lower than the other strains (S4 Fig). The Analysis of Molecular Variance (AMOVA) on these major laboratory strains (ROCK, LVP, and ORL) indicates that significant differentiation exists both between colonies of the same strain and between the strains, with the largest variance explained at the individual level (62.6%), followed by the strain level (27.4%), and the named group (11.5%); S9 Table.

We found no evidence of admixture among the populations on the SNP dataset using the F3-test implemented by TreeMix v. 1.13 [46], as no p-values were significant (P<0.05) following correction for multiple comparisons. The five smallest F3 Statistics, and corresponding p-values, are given in S10 Table. The clustering analysis and the Maximum Likelihood (ML) tree (Figs 3 and 5), suggest that the LVP strain is genetically related to the Asian populations. There is also one colony from ROCK and LVP that does not cluster with the other two colonies from the same strain in the ML tree (Fig 5). The younger strains from Vietnam—HCM and Hanoi—which had been recently colonized (< 20 generations in 4 years), remain genetically close to their originating populations, as can be seen in Figs 3, 4 and 5. The same close relationship was found between the Chetumal population and the Chetumal strain (in colony for ~8 years).

These results demonstrate that colonies of the same laboratory strain acquired from different labs may be significantly divergent. This could be due to either evolution in the lab environment, cross-colony contamination, or mislabeling during rearing, which may be more prevalent than previously thought, with consequences for the repeatability of studies.

### Genetic uniformity of the *Ae*. *aegypti* cell line Aag2 across labs

All 5 Aag2 cell line accessions genotyped for microsatellites were identical at all 12 loci. The genotype concordance of the two Aag2 cell line accessions genotyped for SNPs (Aag2_1 and Aag2_3) was 0.9822, according to the Genotype Concordance tool implemented in Picard v. 2.18.16 [50]. This translates into 15,494 out of 15,776 SNPs shared by these cell lines (excluding missing data). Genetic assignment tests using random sets of ~3,276 SNPs (20% of total SNPs) and the panel of all laboratory and wild populations from this work, identify a ROCK colony (ROCK_Hopkins: 6/10 runs and ROCK_Notre Dame: 2/10 runs) as the closest population matches for this cell line, followed by Siquirres, CR (2/10 runs). The Aag2 cell line is positioned within the clade that contains ROCK_FC and ROCK_Hopkins in the ML tree (Fig 5) and the PCA (Fig 4), positions Aag2 close to the OX513A and the main ROCK cluster (I), as well as in the vicinity of other *Ae*. *ae*. *aegypti* wild strains. Together, this evidence points to minimal genetic changes taking place in the Aag2 cell line over time, regardless of its passage history and laboratory host. This constancy allows for cross-studies comparison and provides a set of genetic markers that can be used for cell line validation, with minimal effort, time investment, and at a relative low cost.

## Discussion

The reproducibility of research findings is an essential part of the scientific method and key for the validation of knowledge. Depending on the type of study, reproducibility may depend on the use of specific biological and/or chemical resources. Since the genetic makeup of an organism strongly affects its phenotype, it is imperative that experiments involving living organisms ensure that their experimental population conforms to the expected genetic description. Examples of laboratory cross-contamination cases that have influenced research outcomes throughout the years include the extensive HeLa cell contamination of human cell lines [8,9,11,54], the cross-contamination of *Caenorhabditis elegans* wild strains with the laboratory adapted Bergerac (N2) strain [1,2], and laboratory contamination of patient cultures with *Mycobacterium tuberculosis* [10]. Laboratory cross-contamination of living stocks, or their evolution can be avoided, minimized, or easily corrected with minimal effort in those stocks amenable to cryopreservation by replenishing them with frozen stocks on a regular basis [55]. When cryopreservation is not an option, reference stock centers may help to preserve the standards to be used by the research community (e.g. the Bloomington Drosophila Stock Center and the Malaria Research and Reference Reagent Resource Center [MR4]). Unfortunately, although MR4 hosts a few strains of *Ae*. *aegypti*, no formal strain repository exists for this or any other *Aedes* vector, and strains altruistically shared among labs often lack documentation. This is likely the reason why the history of *Ae*. *aegypti* strains remains elusive [15], with strains being established, renamed, admixed, or mislabeled on a regular basis without many records.

Confirming the identity of the mosquito populations and cell lines used in each study thus becomes imperative. Here we show that 1) laboratory colonization reduces the genetic diversity of the mosquito population, 2) colonies derived from the same source tend to diverge over time, 3) the origin of the LVP strain used as the genome reference is not *Ae*. *ae*. *formosus*, as suggested by its putative African origin, but rather is genetically related to *Ae*. *ae*. *aegypti* populations from Asia, and 4) the Aag2 *Ae*. *aegypti* cell line has experienced minimal genetic changes within and across the laboratories, likely as a result of proper standard cell culture procedures and their availability at the ATCC repository (USA).

We observed lower genetic diversity in laboratory colonies than in *Ae*. *aegypti* field collections but no significant differences in Ne. A decrease in genetic diversity is likely the result of founder effects and bottlenecks during colony establishment, and can lead to reduction in fitness relative to the ancestral wild population [56], but also to divergent phenotypes [57, 58]. Small and short-term bottlenecks, such as those experienced during the colonization process, strongly impact allelic richness but have little influence on heterozygosity [59]. This is consistent with the diversity estimates measured throughout the colonization process of our two Vietnam populations, where allelic richness declined as a function of generations in the lab, but not the heterozygosity. It is worth noting that heterozygosity in *Ae*. *aegypti* is overall high. Higher than expected levels of heterozygosity in this species are maintained over many generations of lab rearing, likely due to the presence of balanced lethal systems in this species [60, 61].

Bottlenecks and founder effects leading to changes in allele frequencies and the elimination of rare alleles may be in part responsible for the divergence observed among colonies of the same named strain reared in different labs, and the loss of the ancestral genetic signature. However, this alone is unlikely to explain the considerable divergence observed between some of the laboratory strains, such as ROCK, and contamination remains a more viable explanation.

The aforementioned allele frequency changes as a consequence of laboratory colonization would likely be more pronounced in species more challenging to rear in the laboratory. For example, many Neotropical *Anopheles* vectors in which copulation has to be forced or stimulated and day light and temperature conditions require a meticulous control [62, 63, 64], may experience more dramatic losses in genetic diversity compared to the relatively easier to colonize *Ae*. *aegypti* [12].

Low Ne values have been previously reported for *Ae*. *aegypti* populations ([24] and references within), with our data falling within the published estimates. Although we observed a drop in Ne after initial laboratory colonization of the Vietnam strains, the magnitude of such a reduction could be considered minor. The low Ne we observed in the field populations could be explained by a change in census population size due to population bottlenecks (maybe caused by vector control or environmental changes) or founder events. Other parameters influencing Ne in this species may include changes in the number of females successfully obtaining a blood meal and thus contributing to the next generation, a low rate of polyandry [65], short dispersal range (limited migration), and differences in lifespan among males and females.

That decades of laboratory culture have caused both ROCK and LVP strains to become genetically similar to Asian/Australian populations, despite their presumed origins of Cuba and West Africa, respectively, is puzzling (Figs 3–5; [15]). It is tempting to speculate that this could be due to parallel selection under long-term culture, converging on genetic signatures typical of contemporary Asian/Australian populations, favored under the laboratory environment. However, we do not know why this would this be the case.

Mischaracterization or divergence of colonies from the same *Ae*. *aegypti* strain, as evidenced by this study, may lead to misleading phenotypes. For example, ROCK and ORL are known to be susceptible to insecticides and are routinely used as standards in insecticide resistance trials. Similarly, these strains are used in virus competence studies, and genetic variation among colonies may be in part responsible for the broad spectra of results observed (reviewed in [66]). These results suggest a need for *Ae*. *aegypti* strain validation. Establishing a repository for *Ae*. *aegypti* strains, with regular, documented genetic verification, seems warranted, especially in light of the high degree of genetic diversity observed in this mosquito. Alternatively, strains could be validated using the population reference panels that we have generated over time [19,24,26,48], but it would require certain expertise in population genetics. In contrast, validation of the Aag2 *Ae*. *aegypti* cell line can now be achieved using the genotypes produced in this study, similar to human cell line authentication protocols.

## Supporting information

S1 Table Diversity of the two Vietnam strains (Ho Chi Minh [HCM] and Hanoi) based on 12 microsatellites. (DOCX)

S2 Table Effective population size estimated from microsatellites using the single-sample method based on linkage disequilibrium method [29], as implemented in NeEstimator v.2.0 [30]. (DOCX)

S3 Table Effective population size estimated from the SNP dataset using the single-sample method based on linkage disequilibrium method [29], as implemented in NeEstimator v.2.0 [30]. (DOCX)

S4 Table Effective population size estimated from the SNP dataset after removal of first-degree relatives based on output from VCFtools 0.1.14 [35]—relatedness2 command, using the single-sample method based on linkage disequilibrium method [29], as implemented in NeEstimator v.2.0 [30]. (DOCX)

S5 Table Effective population size (Ne) of the two Vietnam strains (HCM and Hanoi). Estimates are from microsatellites following the single-sample method based on linkage disequilibrium (LD), as implemented in NeEstimator v.2.0 [30].
(DOCX)

S6 Table Effective population size (Ne) of the two Vietnam strains (HCM and Hanoi). Estimates are from microsatellites using the two-sample Waples (1989)[31] method and three options for computing the standardized variance in allele frequency, as implemented in NeEstimator v.2.0 [30].
(DOCX)

S7 Table Analysis of variance (ANOVA) on allele frequencies from the two *Aedes aegypti* Vietnam strains, Hanoi and HCM, throughout their colonization process. The star (*) denotes significant values.
(DOCX)

S8 Table Results from the bottleneck analysis of the two *Aedes aegypti* strains from Vietnam sampled through the laboratory colonization, conducted on BOTTLENECK v. 1.2.02. [46]. Significant values are in bold.
(DOCX)

S9 Table Analysis of molecular variance (AMOVA) on allele frequencies from the three major *Aedes aegypti* laboratory strains in this study: Rockefeller (ROCK), Orlando (ORL), and Liverpool (LVP). (DOCX)

S10 Table The five lowest F3 test statistics from the three-population (F3) test in Treemix v. 1.13 [46]. (DOCX)

S1 Fig Effective population size (Ne) estimated from a single population sample using the bias-corrected version of the linkage disequilibrium method from [22], as implemented in NeEstimator v.2.0 [23]. A) using 12 microsatellite markers and B) using 16, 204 SNPs. Dotted lines are the arithmetic mean, dashed lines are the harmonic means.
(TIF)

S2 Fig Genetic structure present among *Ae*. *aegypti* laboratory and wild strains. STRUCTURE bar plots based on 12 microsatellite loci. Each vertical bar represents an individual. The height of each bar represents the probability of assignment to each of K = 3 and K = 18 genetic clusters (different colors). Rockefeller (ROCK); Orlando (ORL), Liverpool (LVP).
(TIF)

S3 Fig Genetic structure present among *Ae*. *aegypti* laboratory and wild strains. LEA v.1.8.1 [44] admixture bar plots based on 16,204 SNPs, after removing first-degree relatives based on output from VCFtools 0.1.14 [35]—relatedness2 command. Each vertical bar represents an individual. The height of each bar represents the probability of assignment to each of K = 3 genetic clusters (different colors). Rockefeller (ROCK); Orlando (ORL), Liverpool (LVP).
(TIF)

S4 Fig Genetic distances (Fst) within and between major *Ae*. *aegypti* strains estimated from 16,204 SNPs, as described by [48] and implemented by the StAMPP package [37] in R v. 3.4.0 [38]. Rockefeller (ROCK); Orlando (ORL), Liverpool (LVP).
(TIF)

S1 File History of *Aedes aegypti* Aag2 cell line and strains. (DOCX)

S2 File Raw genotype calls at 12 microsatellite loci of wild populations and laboratory strains and 5 Aag2 cell line accessions. (TXT)

S3 File Raw genotype calls at 11 microsatellite loci of the two Vietnam strains (Hanoi and Ho Chi Minh) at different generations during the colonization process. (TXT)
