# Supplementary material for: Genetic diversity of laboratory strains and implications for research: The case of Aedes aegypti
Source: PLoS Negl Trop Dis. 2019 Dec 9;13(12):e0007930. doi: 10.1371/journal.pntd.0007930 (PMC6922456; doi:10.1371/journal.pntd.0007930)
Supplement: S1 Table — (DOCX) [file pntd.0007930.s001.docx]

**S1 Table:** Diversity of the two Vietnam strains (Ho Chi Minh [HCM] and Hanoi) based on 12 microsatellites.

| **Population** | **Generation** | **Ho** | **uHe** | **AR(48)** | **PAR(48)** |
| --- | --- | --- | --- | --- | --- |
| HCM | 0 | 0.568 | 0.583 | 4.76 | 1.42 |
| HCM | 4 | 0.463 | 0.484 | 3.72 | 0.08 |
| HCM | 9 | 0.515 | 0.509 | 3.36 | 0 |
| HCM | 16 | 0.460 | 0.459 | 3.28 | 0.02 |
| HCM | 17 | 0.472 | 0.456 | 3.15 | 0 |
| Hanoi | 0 | 0.434 | 0.468 | 3.18 | 0.19 |
| Hanoi | 4 | 0.512 | 0.490 | 3.16 | 0.16 |
| Hanoi | 9 | 0.459 | 0.475 | 2.87 | 0 |
| Hanoi | 15 | 0.521 | 0.533 | 2.93 | 0.07 |
| Hanoi | 16 | 0.510 | 0.531 | 2.96 | 0 |
