# Supplementary material for: Genetic diversity of laboratory strains and implications for research: The case of Aedes aegypti
Source: PLoS Negl Trop Dis. 2019 Dec 9;13(12):e0007930. doi: 10.1371/journal.pntd.0007930 (PMC6922456; doi:10.1371/journal.pntd.0007930)
Supplement: S2 Table — (DOCX) [file pntd.0007930.s002.docx]

**S2 Table:** Effective population size estimated from microsatellites using the single-sample method based on linkage disequilibrium method [29], as implemented in NeEstimator v.2.0 [30].

| **Population** | **Marker** | ***N*** | ***N* Loci** | **Ne (0.02)** | **95% LowCI** | **95% HiCI** |
| --- | --- | --- | --- | --- | --- | --- |
| Hamburg_Strain | micro | 54 | 11 | 37.4 | 19.3 | 98.2 |
| ROCK_Notre Dame | micro | 54 | 11 | 196.7 | 37.7 | 10000 |
| ROCK_FC | micro | 53 | 11 | 66.1 | 36 | 187.3 |
| ROCK_Hopkins | micro | 54 | 12 | 107.1 | 35 | 10000 |
| Oxitec_513A | micro | 54 | 11 | 56.7 | 26.7 | 248.9 |
| LVP_AagL1 | micro | 53 | 6 | 30 | 3.2 | 10000 |
| LVP_WRAIR | micro | 54 | 10 | 107.6 | 40.4 | 10000 |
| Bangkok Strain | micro | 54 | 12 | 69.1 | 27.2 | 10000 |
| D2S3_WRAIR | micro | 54 | 11 | 27.1 | 16 | 51.3 |
| Chetumal Strain | micro | 54 | 11 | 32.6 | 20.3 | 58.5 |
| ORL_CAES | micro | 54 | 10 | 23.3 | 12.5 | 48.4 |
| Surabaya Strain | micro | 54 | 9 | 130.5 | 31.9 | 10000 |
| Hanoi Strain | micro | 48 | 11 | 30.1 | 17.9 | 58.1 |
| Ho Chi Minh Strain | micro | 50 | 12 | 29.8 | 18.9 | 51.9 |
| Key West, FL, USA | micro | 52 | 12 | 20.2 | 15.7 | 26.5 |
| Hanoi_Vietnam | micro | 54 | 12 | 86.8 | 43.9 | 374.7 |
| Ho Chi Minh, Vietnam | micro | 54 | 12 | 75.2 | 48.1 | 143.5 |
| New Orleans, USA | micro | 24 | 12 | 21.5 | 13.8 | 38.1 |
| Tapachula, MX | micro | 54 | 11 | 33.8 | 22.6 | 54.8 |
| Chetumal, MX | micro | 54 | 11 | 21.7 | 15.4 | 31.5 |
| Siquirres, CR | micro | 50 | 12 | 64.4 | 10 | 130 |
| Yaounde, CM | micro | 54 | 12 | 13.4 | 11.3 | 15.8 |
| Lope Forest, GA | micro | 54 | 12 | 82.9 | 59.2 | 129.2 |
| Cairns, AU | micro | 24 | 12 | 13.4 | 8.3 | 23.1 |
| Ouagadougou, BF | micro | 54 | 12 | 14.6 | 12.5 | 16.9 |
| Bangkok, TH | micro | 50 | 11 | 10.1 | 5.1 | 16.9 |
| Houston, TX, USA | micro | 47 | 10 | 7.8 | 3.5 | 14 |
| Patillas, PR | micro | 54 | 12 | 51.4 | 35.7 | 81.5 |

N loci: number of polymorphic loci

*infinite
