# Supplementary material for: Genetic diversity of laboratory strains and implications for research: The case of Aedes aegypti
Source: PLoS Negl Trop Dis. 2019 Dec 9;13(12):e0007930. doi: 10.1371/journal.pntd.0007930 (PMC6922456; doi:10.1371/journal.pntd.0007930)
Supplement: S3 Table — (DOCX) [file pntd.0007930.s003.docx]

**S3 Table:** Effective population size estimated from the SNP dataset using the single-sample method based on linkage disequilibrium method [29], as implemented in NeEstimator v.2.0 [30].

| **Population** | **Marker** | ***Type*** | ***N*** | ***N loci*** | **Ne (0.02)** | **95% LowCI** | **95% HiCI** |
| --- | --- | --- | --- | --- | --- | --- | --- |
| Yaounde, CM | SNP | wild | 16 | 11590 | 3.2 | 3.2 | 3.2 |
| Lope Forest, GA | SNP | wild | 13 | 10813 | 84.3 | 83.4 | 85.3 |
| Cairns, AU | SNP | wild | 12 | 11352 | 2.6 | 2.6 | 2.6 |
| Hanoi, VT | SNP | wild | 22 | 12348 | 18.7 | 18.7 | 18.8 |
| Ho Chi Minh, VT | SNP | wild | 19 | 13685 | 87.5 | 87.1 | 88 |
| Siquirres, CR | SNP | wild | 6 | 12407 | 10000 | 0 | 10000 |
| Tapachula, MX | SNP | wild | 12 | 12118 | 39 | 38.8 | 39.2 |
| Key West, FL, USA | SNP | wild | 12 | 13065 | 9.8 | 9.8 | 9.8 |
| New Orleans, LA,USA | SNP | wild | 12 | 13559 | 12.6 | 12.5 | 12.6 |
| Bangkok_TH | SNP | wild | 11 | 10835 | 6.4 | 6.4 | 6.4 |
| CDC strain | SNP | lab | 20 | 7168 | 14.5 | 14.5 | 14.6 |
| Chetumal strain | SNP | lab | 8 | 11134 | 27.5 | 27.3 | 27.7 |
| Liverpool_AaegL1 | SNP | lab | 10 | 3974 | 6.8 | 6.7 | 6.8 |
| Liverpool_WRAIR | SNP | lab | 8 | 10188 | 30.4 | 30.2 | 30.7 |
| Liverpool_MR4 | SNP | lab | 12 | 8713 | 8.2 | 8.1 | 8.2 |
| Liverpool_AaegL5 | SNP | lab | 7 | 7322 | 3.5 | 3.4 | 3.6 |
| Orlando_FIU | SNP | lab | 12 | 9378 | 13.1 | 13.1 | 13.1 |
| Orlando_PU | SNP | lab | 11 | 8370 | 14.3 | 14.2 | 14.3 |
| Oxitec_513A | SNP | lab | 25 | 9745 | 52.8 | 52.6 | 53 |
| ORL_CAES | SNP | lab | 8 | 9911 | 12.7 | 12.6 | 12.7 |
| ROCK_Hopkins | SNP | lab | 7 | 8079 | 8 | 8 | 8.1 |
| ROCK_FC | SNP | lab | 7 | 11318 | 17.5 | 17.4 | 17.6 |
| ROCK_Notre Dame | SNP | lab | 10 | 10954 | 20.4 | 20.3 | 20.5 |
| NOLA inbred | SNP | lab | 10 | 6094 | 1.5 | 1.5 | 1.5 |
| Surabaya Strain | SNP | lab | 6 | 7693 | 10000 | 0 | 10000 |
| Ho Chi Minh Strain | SNP | lab | 12 | 10911 | 11.5 | 11.5 | 11.6 |
| Hanoi Strain | SNP | lab | 12 | 10519 | 8 | 8 | 8 |

N loci: number of polymorphic loci

*infinite
