# Supplementary material for: Genetic diversity of laboratory strains and implications for research: The case of Aedes aegypti
Source: PLoS Negl Trop Dis. 2019 Dec 9;13(12):e0007930. doi: 10.1371/journal.pntd.0007930 (PMC6922456; doi:10.1371/journal.pntd.0007930)
Supplement: S4 Table — (DOCX) [file pntd.0007930.s004.docx]

**S4 Table:** Effective population size estimated from the SNP dataset after removal of first-degree relatives based on output from VCFtools 0.1.14 [35] --relatedness2 command, using the single-sample method based on linkage disequilibrium method [29], as implemented in NeEstimator v.2.0 [30].

| **Population** | **Marker** | ***Type*** | ***N*** | ***N loci*** | **Ne (0.02)** | **95% LowCI** | **95% HiCI** |
| --- | --- | --- | --- | --- | --- | --- | --- |
| Yaounde, CM | SNP | wild | 10 | 11417 | 32.1 | 31.9 | 32.3 |
| Lope Forest, GA | SNP | wild | 13 | 10813 | 84.3 | 83.4 | 85.3 |
| Cairns, AU | SNP | wild | 6 | 10922 | ∞ | ∞ | ∞ |
| Hanoi, VT | SNP | wild | 18 | 12253 | 25.6 | 25.6 | 25.7 |
| Ho Chi Minh, VT | SNP | wild | 18 | 13663 | 138.4 | 137.1 | 139.7 |
| Siquirres, CR | SNP | wild | 6 | 12407 | ∞ | 0 | ∞ |
| Tapachula, MX | SNP | wild | 12 | 12118 | 39 | 38.8 | 39.2 |
| Key West, FL, USA | SNP | wild | 9 | 12887 | 22.3 | 22.2 | 22.4 |
| New Orleans, LA,USA | SNP | wild | 10 | 13514 | 24.3 | 24.2 | 24.4 |
| Bangkok, TH | SNP | wild | 7 | 10738 | 17.6 | 17.5 | 17.7 |
| CDC strain | SNP | lab | 16 | 7136 | 20.6 | 20.5 | 20.7 |
| Chetumal strain | SNP | lab | 8 | 11134 | 27.5 | 27.3 | 27.7 |
| LVP_AaegL1 | SNP | lab | 5 | 3741 | 169.2 | 138.7 | 216.6 |
| LVP_WRAIR | SNP | lab | 8 | 10188 | 30.4 | 30.2 | 30.7 |
| LVP_MR4 | SNP | lab | 10 | 8701 | 10.3 | 10.2 | 10.3 |
| LVP_AaegL5 | SNP | lab | 2 | 6555 | ∞ | 0 | ∞ |
| ORL_FIU | SNP | lab | 10 | 9150 | 18.4 | 18.3 | 18.5 |
| ORL_PU | SNP | lab | 9 | 8235 | 17.4 | 17.3 | 17.5 |
| Oxitec_513A | SNP | lab | 25 | 9745 | 52.8 | 52.6 | 53 |
| ORL_CAES | SNP | lab | 6 | 9687 | 29.9 | 29.5 | 30.3 |
| ROCK_Hopkins | SNP | lab | 6 | 8014 | 14.2 | 14 | 14.3 |
| ROCK_FC | SNP | lab | 7 | 11318 | 17.5 | 17.4 | 17.6 |
| ROCK_Notre Dame | SNP | lab | 10 | 10954 | 20.4 | 20.3 | 20.5 |
| New Orleans inbred | SNP | lab | 3 | 5456 | ∞ | 0 | ∞ |
| Surabaya strain | SNP | lab | 5 | 7528 | ∞ | 0 | ∞ |
| HCM Strain | SNP | lab | 10 | 10787 | 15.3 | 15.2 | 15.3 |
| Hanoi Strain | SNP | lab | 9 | 10400 | 18.9 | 18.8 | 19 |

N loci: number of polymorphic loci

*infinite
