# Supplementary material for: Genetic diversity of laboratory strains and implications for research: The case of Aedes aegypti
Source: PLoS Negl Trop Dis. 2019 Dec 9;13(12):e0007930. doi: 10.1371/journal.pntd.0007930 (PMC6922456; doi:10.1371/journal.pntd.0007930)
Supplement: S5 Table — Estimates are from microsatellites following the single-sample method based on linkage disequilibrium (LD), as implemented in NeEstimator v.2.0 [30]. (DOCX) [file pntd.0007930.s005.docx]

**S5 Table:** Effective population size (Ne) of the two Vietnam strains (HCM and Hanoi). Estimates are from microsatellites following the single-sample method based on linkage disequilibrium (LD), as implemented in NeEstimator v.2.0 [30].

| **Population** | **Generation** | **Estimate** | **Alleles** | **Ne** | **LowCI** | **HiCI** |
| --- | --- | --- | --- | --- | --- | --- |
| Hanoi | 0 | LD | 54 | 68.5 | 36.9 | 202.5 |
| Hanoi | 4 | LD | 24 | 40 | 16.6 | infinite |
| Hanoi | 9 | LD | 40 | 29 | 16.3 | 63.8 |
| Hanoi | 15 | LD | 40 | 18 | 18.8 | 31.9 |
| Hanoi | 16 | LD | 48 | 30.1 | 17.9 | 58.1 |
| HCM | 0 | LD | 54 | 81.1 | 49.3 | 176 |
| HCM | 4 | LD | 24 | 12.7 | 7.7 | 22.8 |
| HCM | 9 | LD | 40 | 21.3 | 13.4 | 36.6 |
| HCM | 16 | LD | 40 | 10.8 | 3.7 | 10.4 |
| HCM | 17 | LD | 50 | 33.5 | 20.4 | 63.3 |
