# Supplementary material for: Genetic diversity of laboratory strains and implications for research: The case of Aedes aegypti
Source: PLoS Negl Trop Dis. 2019 Dec 9;13(12):e0007930. doi: 10.1371/journal.pntd.0007930 (PMC6922456; doi:10.1371/journal.pntd.0007930)
Supplement: S6 Table — Estimates are from microsatellites using the two-sample Waples (1989)[31] method and three options for computing the standardized variance in allele frequency, as implemented in NeEstimator v.2.0 [30]. (DOCX) [file pntd.0007930.s006.docx]

**S6 Table:** Effective population size (Ne) of the two Vietnam strains (HCM and Hanoi). Estimates are from microsatellites using the two-sample Waples (1989)[31] method and three options for computing the standardized variance in allele frequency, as implemented in NeEstimator v.2.0 [30].

| **Population** | **Estimate** | **Alleles** | **Ne** | **LowCI** | **HiCI** |
| --- | --- | --- | --- | --- | --- |
| Hanoi 00-04 | Pollak | 31 | 50.7 | 21.5 | 138.1 |
| Hanoi 04-09 | Pollak | 28 | 57 | 23.1 | 164.8 |
| Hanoi 09-15 | Pollak | 26 | 36.9 | 17.2 | 73.4 |
| Hanoi 15-16 | Pollak | 27 | 9 | 4.1 | 19.2 |
| HCM 00-04 | Pollak | 45 | 26.4 | 14.5 | 46.8 |
| HCM 04-09 | Pollak | 31 | 85.1 | 32.2 | 363.2 |
| HCM 09-16 | Pollak | 30 | 41.6 | 20.6 | 78.3 |
| HCM 16-17 | Pollak | 28 | 39.5 | 12 | Infinite |
| Hanoi 00-04 | Nei_Tajima | 31 | 48.2 | 20.7 | 126.5 |
| Hanoi 04-09 | Nei_Tajima | 28 | 64.9 | 25.4 | 211.8 |
| Hanoi 09-15 | Nei_Tajima | 26 | 38.5 | 17.8 | 77.4 |
| Hanoi 15-16 | Nei_Tajima | 27 | 12.8 | 5.4 | 31.6 |
| HCM 00-04 | Nei_Tajima | 45 | 26.7 | 14.6 | 47.4 |
| HCM 04-09 | Nei_Tajima | 31 | 83.3 | 31.7 | 342 |
| HCM 09-16 | Nei_Tajima | 30 | 42.4 | 20.9 | 80.2 |
| HCM 16-17 | Nei_Tajima | 28 | 36.4 | 11.4 | 1576.7 |
| Hanoi 00-04 | Jorde_Ryman | 31 | 37.9 | 21.4 | 58.9 |
| Hanoi 04-09 | Jorde_Ryman | 28 | 69.5 | 38 | 110.3 |
| Hanoi 09-15 | Jorde_Ryman | 26 | 36.3 | 19.3 | 58.5 |
| Hanoi 15-16 | Jorde_Ryman | 27 | 14.9 | 8.1 | 23.9 |
| HCM 00-04 | Jorde_Ryman | 45 | 22.9 | 14.5 | 33.3 |
| HCM 04-09 | Jorde_Ryman | 31 | 61.2 | 34.9 | 95.8 |
| HCM 09-16 | Jorde_Ryman | 30 | 36 | 20.2 | 56.4 |
| HCM 16-17 | Jorde_Ryman | 28 | 27.5 | 15.1 | 43.7 |
