# Supplementary material for: Genetic diversity of laboratory strains and implications for research: The case of Aedes aegypti
Source: PLoS Negl Trop Dis. 2019 Dec 9;13(12):e0007930. doi: 10.1371/journal.pntd.0007930 (PMC6922456; doi:10.1371/journal.pntd.0007930)
Supplement: S7 Table — The star (*) denotes significant values. (DOCX) [file pntd.0007930.s007.docx]

**S7 Table:** Analysis of Variance (ANOVA) on allele frequencies from the two *Aedes aegypti* Vietnam strains, Hanoi and HCM, throughout their colonization process. The star (*) denotes significant values.

| **Source of Variation** | **Df** | **Sum Sq** | **Mean Sq** | **F-value** | **Pr(<F)** |
| --- | --- | --- | --- | --- | --- |
| Generation | 1 | 2190.1 | 2190.1 | 5.774 | 0.0473* |
| Population | 1 | 46.6 | 46.6 | 0.123 | 0.7363 |
| Residuals | 7 |  |  |  |  |
