# Supplementary material for: Genetic diversity of laboratory strains and implications for research: The case of Aedes aegypti
Source: PLoS Negl Trop Dis. 2019 Dec 9;13(12):e0007930. doi: 10.1371/journal.pntd.0007930 (PMC6922456; doi:10.1371/journal.pntd.0007930)
Supplement: S8 Table — Significant values are in bold. (DOCX) [file pntd.0007930.s008.docx]

**S8 Table:** Results from the bottleneck analysis of the two *Aedes aegypti* strains from Vietnam sampled through the laboratory colonization, conducted on BOTTLENECK v. 1.2.02. [46]. Significant values are in bold.

|  |  | **IAM** | | **TPM** | | **SMM** | |
| --- | --- | --- | --- | --- | --- | --- | --- |
| **Strain** | **Gen^*^** | **Sign test** | **Wilcoxon^#^** | **Sign test** | **Wilcoxon^#^** | **Sign test** | **Wilcoxon^#^** |
| Hanoi | 0 | 0.08956 | 0.09229 | 0.53710 | 0.90967 | 0.40965 | 0.56934 |
| Hanoi | 4 | **0.00994** | **0.01221** | 0.20844 | 0.10156 | 0.43153 | 0.57715 |
| Hanoi | 9 | **0.00729** | **0.00684** | 0.06495 | 0.08301 | 0.46555 | 0.27832 |
| Hanoi | 15 | **0.00048** | **0.00049** | **0.00108** | **0.00049** | **0.00130** | **0.00049** |
| Hanoi | 16 | **0.00058** | **0.00049** | **0.00124** | **0.00049** | 0.08723 | **0.00244** |
| HCM | 0 | 0.13712 | 0.05225 | 0.36554 | 0.56934 | 0.37666 | 0.38037 |
| HCM | 4 | 0.47043 | 0.4283 | 0.57370 | 1.00000 | 0.19783 | 0.33936 |
| HCM | 9 | **0.02574** | **0.01343** | 0.32511 | 0.17627 | 0.58109 | 0.79102 |
| HCM | 16 | 0.07114 | **0.03418** | 0.25840 | 0.15137 | 0.53304 | 0.73340 |
| HCM | 17 | 0.07092 | **0.04248** | 0.27611 | 0.12939 | 0.54966 | 0.67725 |

^*^Laboratory generation

**^#^** Wilcoxon test (2-tails)

IAM: Infinite allele model

TPM: Two-Phase Model, Variance=36.00, Probability=70.00%, 1000 replications.

SSM: Stepwise Mutation Model
