# Supplementary material for: Genetic diversity of laboratory strains and implications for research: The case of Aedes aegypti
Source: PLoS Negl Trop Dis. 2019 Dec 9;13(12):e0007930. doi: 10.1371/journal.pntd.0007930 (PMC6922456; doi:10.1371/journal.pntd.0007930)
Supplement: S9 Table — (DOCX) [file pntd.0007930.s009.docx]

**S9 Table:** Analysis of Molecular Variance (AMOVA) on allele frequencies from the three major *Aedes aegypti* laboratory strains in this study: Rockefeller (ROCK), Orlando (ORL), and Liverpool (LVP).

| **Source of Variation** | **Nested in** | **% var** | **F-stat** | **F-value** | **Std.Dev.** | **P-value** |
| --- | --- | --- | --- | --- | --- | --- |
| Within Individual | -- | 0.626384 | F_it | 0.373616 | 0.002260 | -- |
| Among Individual | Colony | -0.016315 | F_is | -0.026744 | 0.002028 | 1.000000 |
| Among Colonies | Strain | 0.274246 | F_sc | 0.310123 | 0.001393 | 0.000100 |
| Among Strains | -- | 0.115686 | F_ct | 0.115686 | 0.001705 | 0.024400 |
