# Supplementary material for: Genetic diversity of laboratory strains and implications for research: The case of Aedes aegypti
Source: PLoS Negl Trop Dis. 2019 Dec 9;13(12):e0007930. doi: 10.1371/journal.pntd.0007930 (PMC6922456; doi:10.1371/journal.pntd.0007930)
Supplement: S10 Table — (DOCX) [file pntd.0007930.s010.docx]

**S10 Table**: The five lowest F3 test statistics from the three-population (F3) test in Treemix v. 1.13 [46].

| Sibling Pop ID | Parent 1 ID | Parent 2 ID | F3 Statistic | SE of F3 | Z Score | P-value | Bonferonni adjusted* | Holms adjusted* |
| --- | --- | --- | --- | --- | --- | --- | --- | --- |
| HCM | LVP_AaegL1 | Lope Forest_GA | -0.005 | 0.002 | -2.921 | 0.003 | 1.0 | 0.8 |
| New Orleans | LVP_ AaegL1 | Lope Forest_GA | -0.007 | 0.003 | -2.603 | 0.009 | 1.0 | 1.0 |
| HCM | Surabaya Strain | HCM16 | -0.001 | 0.001 | -2.335 | 0.020 | 1.0 | 1.0 |
| HCM | HCM16 | ROCK_  Notre Dame | -0.001 | 0.001 | -2.238 | 0.025 | 1.0 | 1.0 |
| HCM | HCM16 | ROCK_  Hopkins | -0.001 | 0.001 | -2.136 | 0.033 | 1.0 | 1.0 |

*P-values after correction for multiple comparisons with the method indicated
