# Supplementary figures and images for: Genetic diversity of laboratory strains and implications for research: The case of Aedes aegypti
Source: PLoS Negl Trop Dis. 2019 Dec 9;13(12):e0007930. doi: 10.1371/journal.pntd.0007930 (PMC6922456; doi:10.1371/journal.pntd.0007930)

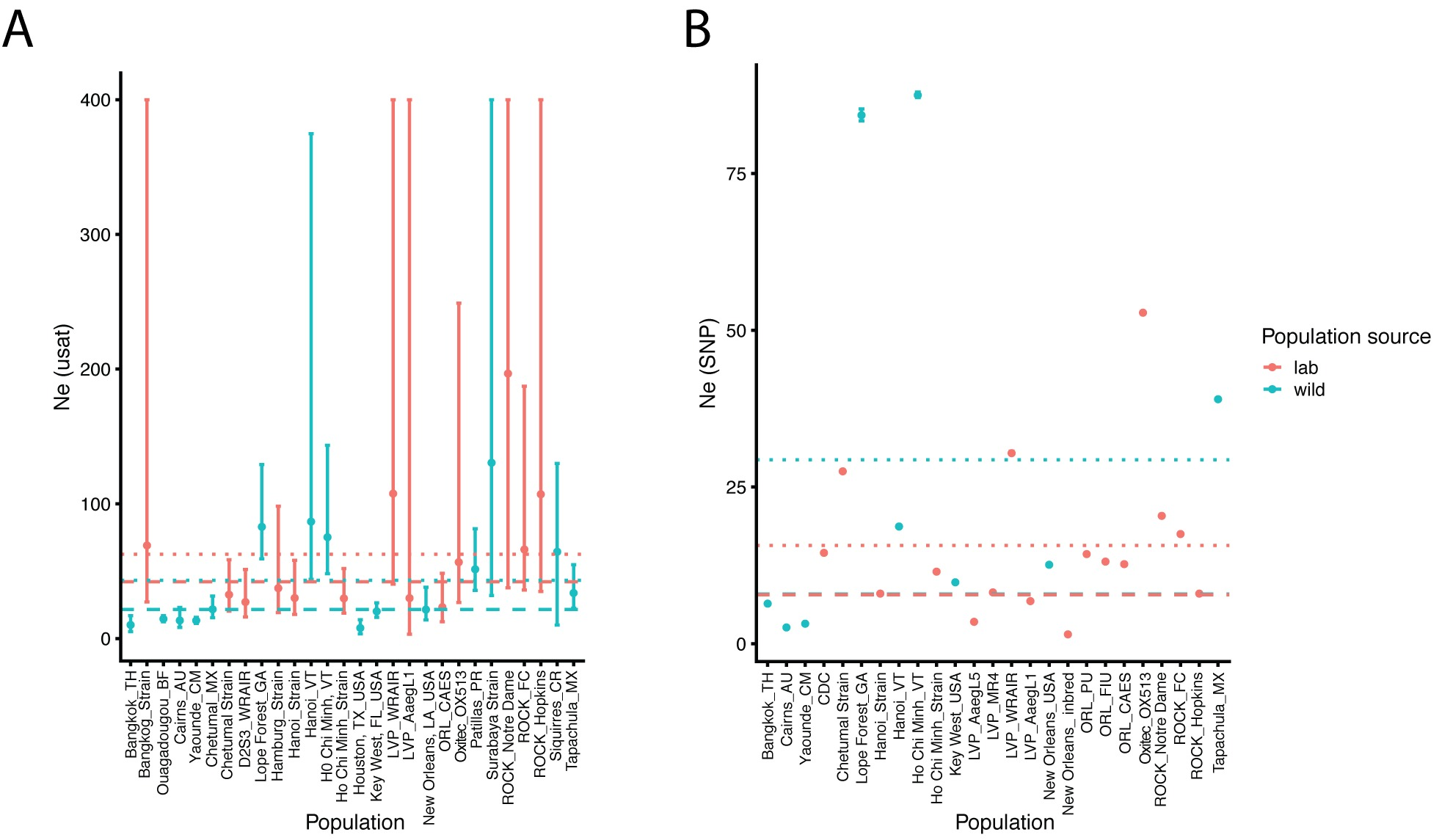

Supplement: S1 Fig — A) using 12 microsatellite markers and B) using 16, 204 SNPs. Dotted lines are the arithmetic mean, dashed lines are the harmonic means. (TIF) [file pntd.0007930.s011.tif]

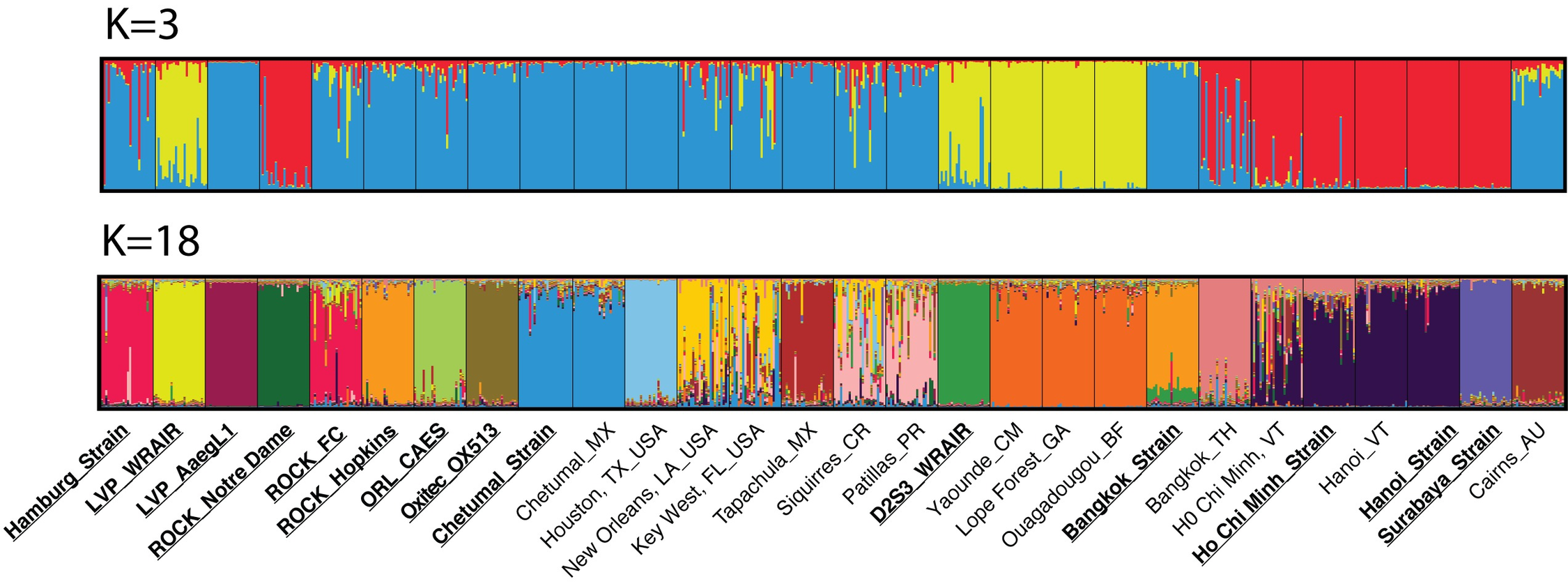

Supplement: S2 Fig — STRUCTURE bar plots based on 12 microsatellite loci. Each vertical bar represents an individual. The height of each bar represents the probability of assignment to each of K = 3 and K = 18 genetic clusters (different colors). Rockefeller (ROCK); Orlando (ORL), Liverpool (LVP). (TIF) [file pntd.0007930.s012.tif]

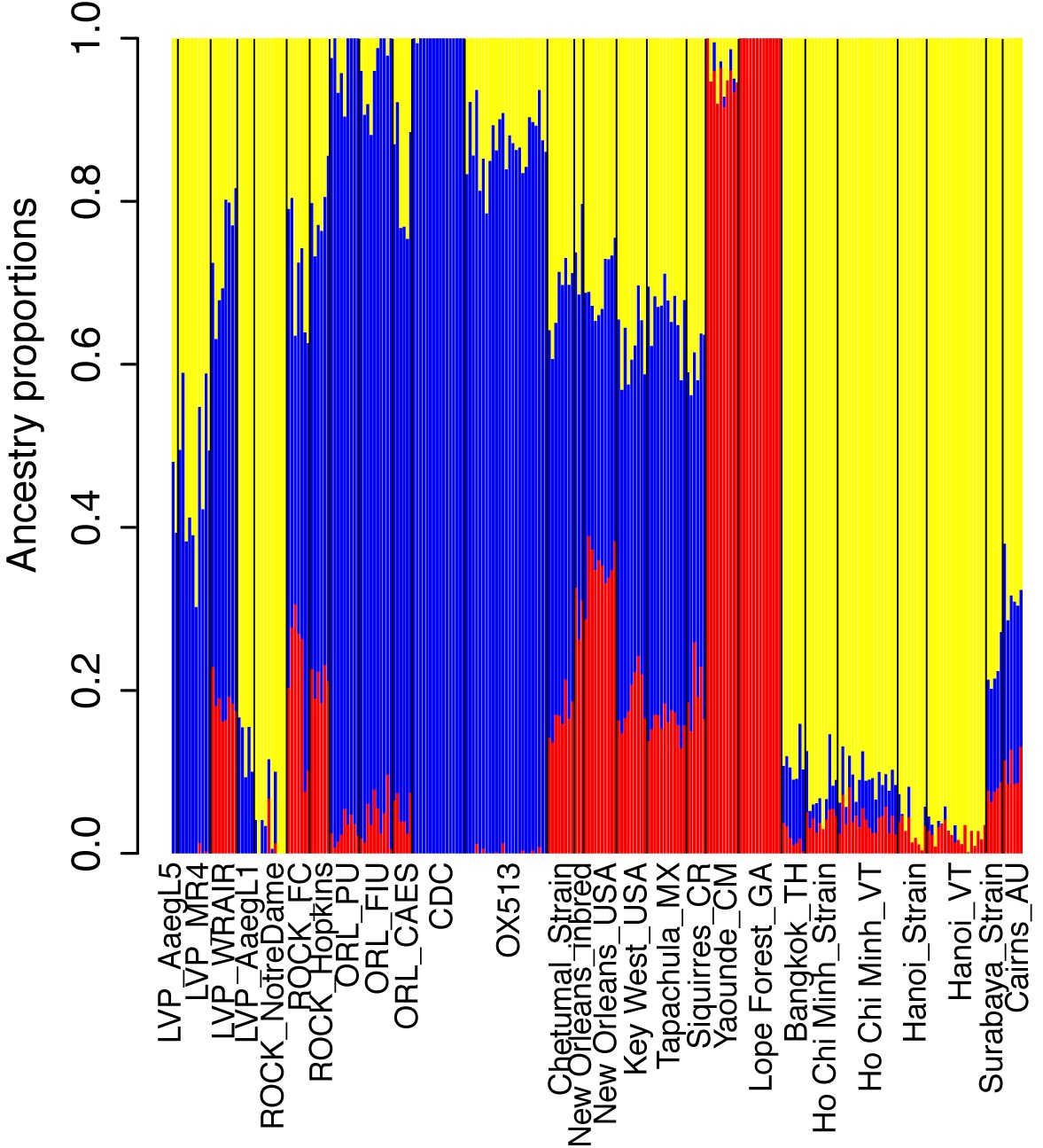

Supplement: S3 Fig — LEA v.1.8.1 [44] admixture bar plots based on 16,204 SNPs, after removing first-degree relatives based on output from VCFtools 0.1.14 [35]—relatedness2 command. Each vertical bar represents an individual. The height of each bar represents the probability of assignment to each of K = 3 genetic clusters (different colors). Rockefeller (ROCK); Orlando (ORL), Liverpool (LVP). (TIF) [file pntd.0007930.s013.tif]

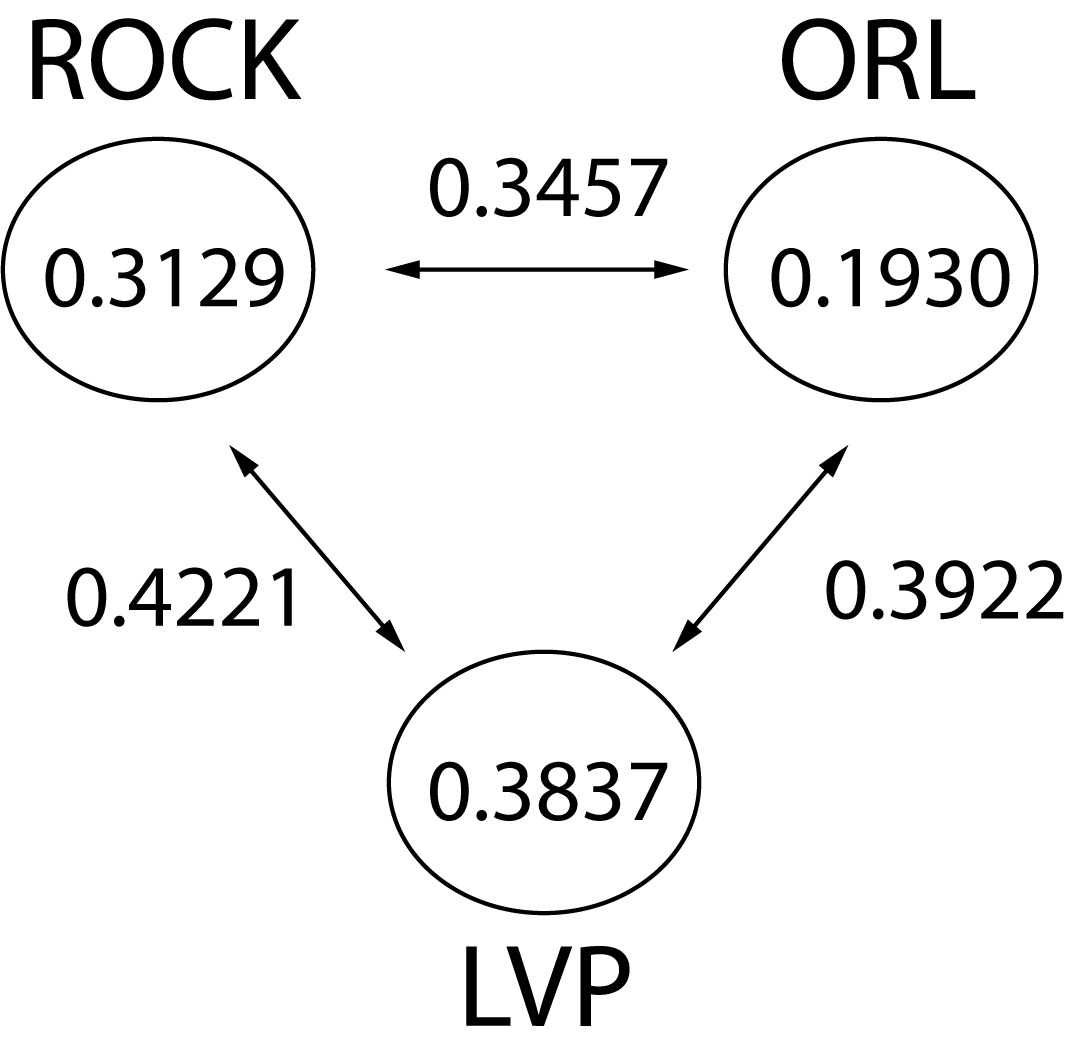

Supplement: S4 Fig — Rockefeller (ROCK); Orlando (ORL), Liverpool (LVP). (TIF) [file pntd.0007930.s014.tif]
