# Supplementary material for: Genetic diversity of laboratory strains and implications for research: The case of Aedes aegypti
Source: PLoS Negl Trop Dis. 2019 Dec 9;13(12):e0007930. doi: 10.1371/journal.pntd.0007930 (PMC6922456; doi:10.1371/journal.pntd.0007930)
Supplement: S1 File — (DOCX) [file pntd.0007930.s015.docx]

History of *Aedes aegypti* AAG2 cell line and strains

**CELL LINE**

**AAG2 cell line**: *Ae. aegypti* cells presumably originated in the laboratory of J. Peleg (Israel Institute for Biological Research, Ness-Ziona, Israel) from embryonic tissue derived from a laboratory colony supplied by Dr. L. Whithman of the Rockefeller Foundation Virus Laboratories (Peleg and Trager, 1963). The cells were recovered from a sample stored in liquid nitrogen since 1976. These cells were subsequently adapted to grow in E-5 medium and were denominated Aag-2 (Lan et al. 1990). We obtained samples from 5 different accessions of this cell line from research laboratories in France, USA (Connecticut, Colorado, and Texas), and Australia.

***Ae. aegypti* STRAINS**

**D2S3 Strain**: “Dengue-2 Susceptible in Chromosome 3” line derived from Bennet et al. 2005. This line was bred from single parent matings of *Ae. aegypti aegypti* (Puerto Rico) and *Ae. aegypti formosus* (Nigeria) and selected to have a high midgut infection rate (MIR) and a high disseminated infection rate (DIR). Selection strategy is described in Bennet et al. (2005). The Puerto Rico (San Juan) mosquitoes were from the 20th laboratory generation. The Nigerian Ibo strain was established from eggs collected in Ibo village, Nigeria (Ballinger-Crabtree et al. 1992) and had been maintained through at least 20 generations in the laboratory. Eggs collected 2013 were used for genotyping.

**Surabaya strain:** Obtained from the Nagasaki University in 2002. Originally collected at Surabaya Indonesia in 2000 (Tsuda et al. 2003). It was believed to be *Ae. queenslandensis*, a third described subspecies of *Aedes aegypti* (Mori et al. 2016). Subsequent work by us [19] and Rašić, et al. (2016) confirmed that this strain was part of the Asian clade of *Ae. aegypti aegypti*. Genotyped individuals were collected in 2013 after undergoing 7 generations of selection for dense tergal abdominal scaling (Mori et al. 2016).

**New Orleans_Inbred:** Colony established from eggs collected in New Orleans in 2015 and taken through seven generations of single-pair mating by J. Powell at Yale University (described in Powell and Evans, 2017).

**Rockefeller (ROCK):** Originally derived from Habana, Cuba (1901) this colony have passed through many laboratories and likely several hundreds of generations. The strain was originally used at the Carlos Finlay Institute in Havana and shipped to the Department of Tropical Medicine at Harvard University in 1926 [15]. In 1935, eggs were passed to the Department of Animal and Plant Pathology of the Rockefeller Institute of Medical Research at Princeton, NJ. In 1936, was shipped to the Rutgers University. In 1958, arrived to Fort Detrick, MD. In 1959, was shipped from Fort Detrick to the University of Notre Dame. More details on the history of this strain are provided in [15].

**ROCK_Notre Dame.** D. Severson lab. University of Notre Dame strain.

**ROCK_FC.** Fio Cruz strain. Received around 1999 from a laboratory in Sao Paulo that received them originally from the CDC, USA.

**ROCK_Hopkins.** Received from G. Dimopoulos lab John Hopkins University) that obtained it from A. Raikhel’s lab (UC Riverside), according to Z. Xi, between 2006-2007.

**Oxitec_OX513A:** RIDL (Release of Insects carrying a Dominant Lethal) strain of *Ae. aegypti*. It has a single transgenic sequence encoding a red fluorescent marker and tetracycline-repressible late-acting dominant lethality (Phuc et al. 2007)*.* Constructed in 2002 from a ROCK strain obtained from Roger Wood, University of Manchester. The strain was transformed by standard microinjection into adult mosquitoes. The strain was made homozygous by repeated backcrossing and then the insert was introgressed into a strain from the Instituto Nacional de Salud Pública (INSP), Mexico (probably from Tapachula, Chiapas) by five generations of backcrossing. For more details about the strain see Phuc et al. 2007; Harris et al. 2011; Bargielowski et al. 2011). Oxitec has maintained the line as a continuously cycling insect colony for the equivalent of over 100 generations. (Harris et al. 2011; Bargielowski et al. 2011).

**Orlando (ORL):** Origin unclear. Strain used by the Bureau of Entomology and Plant Quarantine of USDA in Orlando, FL (1939-1942). May have been mixed with “ROCK” at the Rutgers University to “strengthen” the former strain. The “Orlando” strain was regularly supplemented with wild mosquitoes until circa 1992. The origin of the “wild mosquitoes” used to supplement is unknown, but they may have been local (Florida)[15].

**ORL_PU.** Princeton University. Received from Rob Harrell (University of Maryland) in 2015.

**ORL_FIU.** Florida International University.

**ORL_CAES.** The Connecticut Agricultural Experiment Station strain. Originally established in 1952, has been in continuous culture in the insectary of the Mosquito and Fly Research Unit at the Center for Medical, Agricultural, and Veterinary Entomology (CMAVE), USDA-ARS, Gainesville. The colony is passed approximately every 2-3 weeks (~ 20 generations a year x 60 years), we estimate that it has been colonized for ~ 1200 generations. The CAES colony was received between 2002 or 2004 (personal communication Andreadis; April 2014).

**Liverpool (LVP):** Origin unclear. Described as of West African origin [18], with Freetown, Sierra Leone as the most likely source [15]. The Liverpool strain sequenced for the *Ae. aegypti* genome project AaegL1-3 had been maintained at the Liverpool School of Tropical Medicine since 1936. It was selected for susceptibility to the filarial worm parasite *Brugia malayi*, and subsequently a substrain (LVP^sbm^) was selected for greater *Brugia* susceptibility. Genome sequencing was performed on DNA purified from newly hatched larvae of an LVP^sbm^ strain, which was derived after 12 consecutive generations of single pair inbreeding. This inbred strain has been designated *Ae. aegypti* LVP^1b12^ [*www.vectorbase.org* and 21]. The *Aedes aegypti* Liverpool AGWG (LVP_AGWG) strain was most recently sequenced as part of the *Ae. aegypti* Genome Working Group (AGWG) effort to improve the existing Liverpool strain assembly [17]. The LVP_AGWG strain was generated from 3 generations of single pair inbreeding from inbred sub-strain LVP^IB12^ (source Virginia Tech - VT). DNA sequencing was performed upon a pool of 80 whole male pupae siblings.

**LVP_AaegL5.** Vosshall lab (LVPIB12-VT_Vosshall): Used by *Ae. aegypti* Genome Working Group to generate the AaegL5 assembly.

**LVP_MR4.** The Malaria Research and Reference Reagent Resource Center (LVPib12_MR4)

**LVP_WRAIR.** Walter Reed Army institute of Research (WRAIR): Adults from eggs collected in 2013 were genotyped.

**LVP_AaegL1**. D. Severson lab, U. Notre Dame used to generate the AaegL1 assembly.

**Chetumal strain:** Derived from mosquitoes collected in Chetumal (Yucatan Peninsula, Mexico) in 2005 (Bernhardt et al. 2012). The colony is maintained at Walter Reed Army institute of Research (WRAIR). Adults from eggs collected 2013 were genotyped.

**CDC:** Known as MRA-726, was originally derived from Costa Rica, the colony was established by M. J. Perich in 2001 in Puntarenas, Costa Rica, South America (Perich et al. 2003). The strain was obtained through BEI Resources and kept at the Alameda CDC facility in California (generation F32 onwards).

**Bangkok Strain:** Unclear origin, putatively from Bangkok and named “BAK” before arriving at the Powell lab around 2012.

**Hamburg Strain**: Origin unclear but suggested to have derived from a Liverpool Strain kept at the Institute for Tropical Medicine in Hamburg.

**Vietnam strains (HCM and Hanoi)**: Eggs from the first laboratory generation (F1) of *Ae. aegypti* collected in Ho Chi Minh City and Hanoi, Vietnam, were send to the Powell laboratory in October 2013 by D. Gubler (Duke U.), where they were used to start the HCM and Hanoi strains, respectively. The strains have undergone an average of 4 generations per year. Population samples collected during the colonization process and stored at -20^o^C for further analysis.

Relevant references:

- Bargielowski, I., Nimmo, D., Alphey, L. and Koella, J.C., 2011. Comparison of life history characteristics of the genetically modified OX513A line and a wild type strain of Aedes aegypti. *PLoS One*, *6*(6), p.e20699.
- Bennett, K.E., Beaty, B.J. and Black IV, W.C., 2005. Selection of D2S3, an Aedes aegypti (Diptera: Culicidae) strain with high oral susceptibility to Dengue 2 virus and D2MEB, a strain with a midgut barrier to Dengue 2 escape. *Journal of medical entomology*, *42*(2), pp.110-119.
- Bernhardt SA, Simmons MP, Blair CD, Olson KE, Beaty BJ, et al. (2012) Rapid intraspecific evolution of miRNA and siRNA genes in the mosquito Aedes aegypti. PLoS One, e44198.
- Lan, Q. and Fallon, A.M., 1990. Small heat shock proteins distinguish between two mosquito species and confirm identity of their cell lines. *The American journal of tropical medicine and hygiene*, *43*(6), pp.669-676.
- Mori A, Tsuda Y, Takagi M, Higa Y, Severson DW. Multiple QTL determine dorsal abdominal scale patterns in the mosquito *Aedes aegypti*. Journal of Heredity. 2016 Apr 29;107(5):438-44.
- Peleg, J. and Trager, W., 1963. Cultivation of insect tissues in vitro and their application to the study of arthropod-borne viruses. *The American journal of tropical medicine and hygiene*, *12*(5), pp.820-824.
- Perich, M. J., Rocha O., Castro, L., Alfaro W., Platt K.B., Solano T., Rowley W.A. (2003). Evaluation of the Efficacy of Lambda-Cyhalothrin Applied by Three Spray Application Methods for Emergency Control of *Aedes aegypti* in Costa Rica. J. Am. Mosq. Control Assoc. 19 (2003): 58-62. PubMed: 12674536.
- Phuc, H.K., Andreasen, M.H., Burton, R.S., Vass, C., Epton, M.J., Pape, G., Fu, G., Condon, K.C., Scaife, S., Donnelly, C.A. and Coleman, P.G., 2007. Late-acting dominant lethal genetic systems and mosquito control. *BMC biology*, *5*(1), p.11.
- Powell, J.R. and Evans, B.R., 2017. How much does inbreeding reduce heterozygosity? Empirical results from Aedes aegypti. *The American journal of tropical medicine and hygiene*, *96*(1), pp.157-158.
- Tsuda, Y., Yoopranoto, S., Bendryman, S.S., Rosmanida, R., Dachlan, Y.P. and Takagi, M., 2003. Seasonal changes in variation of dorsal scale pattern of Aedes aegypti (L.)(Diptera: Culicidae) in Surabaya, Indonesia. *Medical Entomology and Zoology*, *54*(1), pp.73-80.
